# Maternal reproductive precocity shapes immunometabolic responses to lipopolysaccharide challenge in Japanese quail offspring

**DOI:** 10.1016/j.psj.2026.107553

**Published:** 2026-08-03

**Authors:** Juliana dos Santos Conceição, Eliane Gasparino, Angélica de Souza Khatlab, Marcos Adriano Pereira Barbosa, Vanicleide da Silva Santos, Lucas Soares da Silva, Marisa Silva Bastos, Thais Pacheco Santana, Simara Márcia Marcato, Emine Mayer Chamse Ddine, Vittor Tuzzi Zancanella, Claudson Oliveira Brito, Jodnes Sobreira Vieira, Ana Paula del Vesco

**Affiliations:** a State University of Maringá, Maringá, Paraná, Brazil; b Federal University of Sergipe, São Cristóvão, Sergipe, Brazil; c Federal University of Alagoas, Arapiraca, Alagoas, Brazil

**Keywords:** Endotoxin challenge, Immunometabolism, Intestinal transcriptomics, Maternal programming

## Abstract

Productive efficiency in laying breeders is influenced by genetic and physiological factors, but it remains unclear whether maternal reproductive precocity programs distinct inflammatory responses in the offspring. This study evaluated the effects of maternal precocity on productive performance, immunological parameters, and the intestinal transcriptomic profile of Japanese quail offspring challenged with lipopolysaccharide (LPS). A design with two maternal groups was used (early-maturing: reaching 95% lay between weeks 14-15; and late-maturing: between weeks 17-18), and the offspring were either challenged or not with LPS at 25 days of age, with jejunum collection for RNA sequencing. Early-maturing dams showed better productive performance and lower stress indicators (lower heterophil:lymphocyte ratio and lower relative spleen weight) (p < 0.05). Their offspring, when challenged with LPS, exhibited a metabolic suppression profile, with downregulation of genes linked to oxidative phosphorylation (*NDUFA5, NDUFS6*; adj. p = 0.006 for the oxidative phosphorylation pathway), NADH metabolism (*GPD1*; adj. p = 0.005 for NADH metabolic process), and amino acid transport (*SLC25A13, SLC25A15*; adj. p = 0.005 for l-amino acid transmembrane transport), associated with activation of anti-inflammatory mediators (*IL18BP, IL1R2*; adj. p = 0.032 for cytokine binding), characterizing an energy-saving tolerance strategy to inflammation. In contrast, the offspring of late-maturing dams challenged with LPS showed strong activation of matrix metalloproteinase *MMP7* (adj. p = 0.004 for collagen catabolic process) and exclusive enrichment of the Wnt signaling pathway (adj. p = 0.003), indicating a response directed toward extracellular matrix remodeling and tissue repair. In conclusion, maternal precocity programs two distinct transcriptional phenotypes in the offspring facing an inflammatory challenge: metabolic tolerance with immunomodulation (descendants of early-maturing dams) versus resistance with tissue remodeling (descendants of late-maturing dams), demonstrating that maternal genetic merit determines different resource allocation strategies between immune defense and homeostasis maintenance.

## Introduction

Productive efficiency in laying birds results from the interaction among genetic, physiological, and environmental factors, and the reproductive precocity of breeders is one of the most relevant traits for performance throughout the production cycle (Francoeur et al., 2025). In Japanese quails, the anticipation of sexual maturity has been associated with greater laying persistence and better feed efficiency, reflecting differences in the allocation of energetic resources and in metabolic regulation (Arbaiza-Bayona *et al*., 2022).

Maternal programming is traditionally understood as the process by which the female’s physiological conditions during reproduction affect embryonic development and offspring phenotype, often through components deposited in the egg, such as hormones, metabolites, and immune factors (Angove and Forder, 2020; De Haas *et al*., 2021). However, when the rearing environment is standardized, differences in progeny performance may reflect not only the dam’s momentary physiological state but, more fundamentally, her genetic merit. In birds, there is evidence indicating that maternal genetic constitution can influence the early growth, metabolic efficiency, and immune response of the offspring independently of the environment (Widowski *et al*., 2022).

In this context, female precocity can be used as an indicator of genetic superiority, and understanding the molecular mechanisms by which the maternal genotype modulates gene expression and the physiological responses of descendants in the face of environmental challenges is an essential step toward advancing beyond what we already know.

Among the biological models available to investigate these interactions, the inflammatory response stands out because it involves intense metabolic reprogramming and coordinated activation of immune pathways. Coupled with this perspective, the use of experimental challenges, such as the administration of lipopolysaccharide (LPS), allows the controlled evaluation of the innate immune response and its impacts on metabolism and performance. LPS, a component of the outer membrane of Gram-negative bacteria, is widely employed to induce systemic inflammation and to investigate stress-response mechanisms in birds, including changes in growth, feed efficiency, and the activation of molecular pathways associated with immunity (Cheng *et al*., 2026).

Given this, the hypothesis is raised that the reproductive precocity of dams, as a trait associated with physiological and reproductive potential, may program different response strategies to the inflammatory challenge in the progeny, reflected both in phenotypic characteristics and in transcriptomic profiles. Thus, the objective of this study was to evaluate the effects of maternal precocity on the productive performance, immunological parameters, and transcriptomic profile of Japanese quail offspring subjected to an LPS challenge, aiming to integrate phenotypic and molecular responses for a better understanding of the mechanisms involved in maternal programming.

## Methodology

The experiment was conducted at the Iguatemi Experimental Farm, State University of Maringá, Maringá, Paraná, Brazil. All procedures were approved by the local Animal Ethics Committee (protocol No. 8954270323).

### Dams

Two hundred female Japanese quails (*Coturnix coturnix japonica*) were raised under conventional management from the first day of life until 98 days of age. During this period, the body development of the birds and the laying rate were continuously monitored. At 98 days, the quails were classified based on productive performance into two experimental groups: early-maturing dams (reaching 95% lay between the 14th and 15th week; n = 50) and late-maturing dams (reaching 95% lay between the 17th and 18th week; n = 50).

Over the 35-day experimental period (14th to 19th week), egg production was recorded daily. Productive performance parameters were also evaluated, such as feed conversion per egg mass (FC/EM), feed conversion per dozen eggs (FC/DE), total number of eggs produced, laying rate (%), and egg mass (g), according to the methodology described by Bastos *et al*. (2017).

At 119 days of age, the mating period began for all females under evaluation, using the ratio of one male to two females. The males were previously standardized for body weight and kept in daily contact with the dams for a one-hour period. To minimize possible paternal effects, a rotation system was employed among the males. In the following week, when the dams reached 126 days of age, hatching eggs were collected over a consecutive 7-day interval.

The collected eggs were duly identified, individually weighed, and stored under controlled temperature of 16°C and relative humidity of 78%. Throughout the experiment, the dams were kept in individual cages, with free access to water and a feed formulated to meet the nutritional requirements of the species, according to the recommendations of Rostagno *et al*. (2011), containing 2899 kcal/kg metabolizable energy, 20.7% crude protein, 3.151% calcium, and 0.33% available phosphorus.

The lighting program was kept constant throughout the experimental period, with a daily photoperiod of 17 hours of light. After the end of egg collection, the dams were euthanized by cervical dislocation, and the spleen weight of the dams was evaluated.

### Dams’ heterophils and lymphocytes

To evaluate the heterophil-to-lymphocyte ratio, a blood smear was prepared. A 10 µL aliquot of blood was placed on a microscopy slide and, with the aid of another slide (spreader), a smooth backward motion was performed, allowing the drop to distribute evenly over the surface. The slides were left to air-dry and subsequently stained according to the instructions of the hematological staining kit (Instant Prov, Newprov). Then, 100 cells, including heterophils and lymphocytes, were counted under a microscope to determine the ratio between these two cell types.

### Incubation and embryonic development

On the last day of collection, all eggs from the dams of both groups were previously adapted to ambient temperature and placed in plastic fruit nets, preserving the individual identification of maternal origin. The eggs were then incubated at 37.8°C and 60% relative humidity in an automatic incubator (Chocmaster Luna 240).

During the incubation period, the eggs were positioned horizontally and subjected to automatic turning every two hours until the 15th day. The hatching process was monitored every three hours, starting on the 16th day of incubation (384 hours) and extending until the 21st day (504 hours).

### Progeny

The physical quality of all newly hatched chicks was assessed by measuring body weight and chick length, as well as analyzing general physical aspects, according to the scoring system proposed by Tona *et al*. (2003). After evaluation, the chicks were individually identified and housed according to the experimental group of the respective dam.

The chicks were kept in previously heated brooder rings with heating lamps, with *ad libitum* access to water and a starter feed formulated according to Rostagno *et al*. (2011), containing 2899 kcal/kg metabolizable energy and 20.7% crude protein. The birds were raised under conventional management until 25 days of age. For each chick, the initial weight (1 day of age), final weight (25 days of age), and feed conversion were evaluated.

At 25 days of age, the birds descending from each dam were weighed and distributed into two treatments related to the environmental challenge: birds subjected to inoculation with lipopolysaccharide (LPS) and birds not subjected to inoculation (SLPS).

The birds in the LPS group received an intraperitoneal injection containing 100 µL of LPS from *Escherichia coli* O111:B4 (Sigma-Aldrich, Brazil), at a dose of 100 µg kg⁻¹ body weight. The birds in the unchallenged group received 100 µL of sterile saline solution intraperitoneally. The 3.5-hour post-inoculation interval was selected to capture the acute phase of the inflammatory response, when activation of innate immune pathways and early transcriptional changes are expected to be most pronounced. Similar sampling intervals (3–4 h after LPS challenge) have been successfully used in poultry studies evaluating acute immune and transcriptomic responses (Nunes et al., 2025; Yang et al., 2025; Conceição et al., 2026). Three and a half hours after inoculation, all birds were euthanized by cervical dislocation for the collection of biological samples. Heart weight, spleen weight, and their respective relative weights were evaluated and calculated as: (organ weight/bird weight) × 100.

### Statistical analysis of phenotypic data

The obtained data were first evaluated for normality and homogeneity of variances using the Shapiro-Wilk and Bartlett tests, respectively. When the statistical assumptions were met, the data were subjected to analysis of variance (ANOVA) in a two-factor factorial scheme, considering the effects of dam group, LPS challenge, and the interaction between these factors.

As no significant interactions between factors were observed, treatment means were compared using the t-test, adopting a significance level of 5% (p < 0.05). All statistical analyses were performed in the R software, version 4.5.0.

### RNA sequencing

RNA sequencing was conducted from jejunum fragments of Japanese quails at 25 days of age, using four biological replicates per treatment. The comparisons were performed as follows: (i) offspring from early-maturing dams subjected to the LPS challenge versus offspring from early-maturing dams without the LPS challenge; and (ii) offspring from late-maturing dams subjected to the LPS challenge versus offspring from late-maturing dams without the LPS challenge.

### mRNA preparation

Total RNA was extracted from the jejunum samples using Trizol® reagent (Invitrogen, Carlsbad, CA, USA), at a ratio of 1 mL per 100 mg of tissue, strictly following the manufacturer’s instructions. RNA quantification was performed by spectrophotometry at 260 nm, and sample integrity was assessed by 1% agarose gel electrophoresis under ultraviolet light. Total RNA samples with adequate quality were subjected to next-generation sequencing on the Illumina platform.

### Quality control, mapping, and quantification of expression

The sequencing reads were subjected to initial processing using the BBDuk software, part of the BBTools package version 39.06 (Bushnell, 2023), for the removal of Illumina adapters and low-quality bases. This step aimed to reduce technical noise and minimize possible errors in subsequent analyses.

After quality control, the sequences were mapped against the reference genome using the STAR aligner version 2.7.11b (Dobin *et al*., 2013), allowing the identification of the genomic origin of the reads and their association with specific genes. Gene-level quantification of reads was performed with the featureCounts software version 2.0.8 (Liao, Smyth and Shi, 2014), generating estimates of the expression level of each gene.

The reference genome and annotation file used corresponded to the version GCF_001577835.2_Coturnix_japonica_2.1 (Morris *et al*., 2020), available in the NCBI repository.

### Differential expression analysis

Differential gene expression analysis was conducted from gene-level read counts using the DESeq2 package version 1.42.0 (Love, Huber and Anders, 2014), available in the R/Bioconductor environment (Gentleman *et al*., 2004; Huber *et al*., 2015).

Initially, the counts from each library were used to estimate normalization factors related to sequencing depth per sample, as well as data dispersion, assuming a negative binomial distribution. Next, the data were modeled using a generalized linear model fitted with the quasi-likelihood method (Lund *et al*., 2012). An independent filtering procedure was applied to reduce the number of multiple tests and increase the statistical power of the analysis (Bourgon, Gentleman and Huber, 2010). The evaluation of differential expression for the contrasts of interest was performed using the Wald test.

The obtained p-values were adjusted by the false discovery rate (FDR) method, as proposed by Benjamini and Hochberg (1995), aiming to control false positives. Genes with FDR < 0.05 were considered differentially expressed.

### Functional analysis of differentially expressed genes (DEGs)

Functional analysis of the DEGs was performed using the clusterProfiler package version 4.5.0 (Yu *et al*., 2012) in the R environment (R Core Team, 2023), employing the org.Gg.eg.db annotation database (Carlson, 2019), referring to the species *Gallus gallus*. Gene symbols were converted to Entrez identifiers using the bitr() function, ensuring compatibility with functional enrichment tools and standardization of annotations.

Gene Ontology (GO) enrichment analysis was conducted considering simultaneously the three main domains—biological processes, molecular functions, and cellular components—through the parameter ont = "ALL". Adjustment for multiple comparisons was performed using the Benjamini-Hochberg method (pAdjustMethod = "BH"). Significantly enriched terms were identified by adopting cut-off values of adjusted p (pvalueCutoff = 0.05) and q-value (qvalueCutoff = 0.05). The readable = TRUE option was used to convert Entrez IDs into gene symbols recognized by the Vertebrate Gene Nomenclature Committee (VGNC) (Jones *et al*., 2023), facilitating the biological interpretation of the results.

The results were organized into a dataframe, with the GO terms ranked according to statistical significance (−log10(FDR)), allowing the identification of the most relevant pathways. Visualization was performed using the dotplot() function from the enrichplot package, highlighting the most enriched terms in each GO domain. Additionally, the relationships among the enriched terms were explored using the emapplot() function, based on semantic similarity (pairwise_termsim), while the cnetplot() function was used to visualize the connections between genes and biological pathways, emphasizing the terms with the lowest p-values.

### Functional analysis of DEGs in the Kyoto Encyclopedia of Genes and Genomes (KEGG)

Enrichment analysis of metabolic and signaling pathways was performed using the DEGs previously identified by RNA sequencing, considering genes with adjusted p < 0.05 as significant. This criterion was adopted to ensure the inclusion of genes with statistically robust and biologically relevant changes.

To ensure compatibility with the KEGG database, the gene symbols were converted to Entrez identifiers using the org.Gg.eg.db package specific to *G. gallus*. Genes that did not have a correspondence in the database were excluded from the analysis. Pathway enrichment was conducted using the clusterProfiler package, applying Benjamini-Hochberg correction for multiple tests. Only pathways containing between 10 and 500 genes were considered, avoiding the inclusion of overly broad or overly restricted pathways.

To facilitate biological interpretation, the Entrez IDs were converted back into gene symbols recognized by the VGNC (Jones *et al*., 2023). Redundancy among overlapping pathways was reduced through consolidation and simplification procedures, allowing a clearer and more integrated analysis of the biological processes involved.

### Gene Set Enrichment Analysis (GSEA)

To complement the analysis of differentially expressed genes (DEGs) and to identify coordinated changes in biological pathways that do not reach statistical significance at the single-gene level, Gene Set Enrichment Analysis (GSEA) was performed. This analysis was conducted using the fgsea package (version 1.28.0) in the R statistical environment (version 4.5.0) (Korotkevich *et al*., 2021).

A ranked gene list was generated from the complete differential expression results of DESeq2. For each gene, a ranking score was calculated using the Wald statistic (stat) provided by DESeq2. The genes were then ordered in descending order based on this score, generating a pre-ranked list containing all genes expressed in the jejunum transcriptome.

The reference gene sets (pathways) were obtained from the Molecular Signatures Database (MSigDB) (Subramanian *et al*., 2005; Liberzon *et al*., 2015), specifically the KEGG canonical pathways collection (CP:KEGG_LEGACY) for *Gallus gallus*. To ensure statistical robustness, only gene sets containing between 10 and 500 genes were considered in the analysis.

Gene set enrichment was assessed through 10,000 permutations to estimate statistical significance. The Type I Error (false positives) resulting from multiple tests was controlled by calculating the False Discovery Rate (FDR) using the Benjamini-Hochberg method. Pathways with FDR < 0.05 were considered significantly enriched. The direction of enrichment was determined by the Normalized Enrichment Score (NES), where positive values indicate enrichment of the pathway in the LPS-treated group, while negative values indicate suppression of the pathway in the same group relative to the control.

## Results and discussion

### Performance of dams and progeny

The dams classified as early-maturing showed a higher number of eggs (33.7 *vs.* 25.3; p <.00001) and a higher laying rate (96% *vs.* 72%; p <.00001) compared to late-maturing dams (Table 1). Early-maturing dams also presented greater egg mass (374 *vs*. 271 g/bird) and better feed efficiency, evidenced by lower values of feed conversion per egg mass (2.46 *vs.* 3.35; p <.00001) and per dozen eggs (2.83 *vs*. 3.70; p = 0.00014) compared to those from late-maturing dams.

**Table 1:** Productive performance of Japanese quail breeding stock classified as early-maturing or late-maturing.

Dams	NE*	EM (g/day/bird)	FCR/egg	FCR/egg/35 days	FCR/dozen eggs	Laying rate
Early	33.690^a^ ±3.25	374.080^a^ ±49.7	3.097^b^ ±0.59	2.463^b^ ±0.47	2.833^b^ ±0.41	96.256^a^ ±9.29
Late	25.316^b^ ±4.11	270.895^b^ ±53.0	4.211^a^ ±1.39	3.350^a^ ±1.11	3.706^a^ ±1.11	72.331^b^ ±11.7
p-value	<,00001	<,00001	0.00014	0.00014	0.00014	<,00001

NE*: number of eggs; EM: Egg mass (g/day/bird); FCR/egg: Feed conversion ratio per egg; FCR/egg/35 days: Feed conversion ratio per egg at 35 days; FCR/dozen eggs: Feed conversion ratio per dozen eggs; ^a,b^Different letters in the same column indicate statistical significance based on the t-test with a p-value <0.05.

The results of the present study indicate that reproductive precocity in Japanese quails is associated with consistent improvements in productive performance, including a higher number of eggs, a higher laying rate, and better feed efficiency. These findings are in agreement with studies in production birds showing that the anticipation of sexual maturity may be related to greater laying persistence and better metabolic efficiency throughout the productive cycle (Robinson *et al*., 2001; Tan *et al*., 2021). However, although this association is consistent, the underlying mechanisms are not yet completely elucidated.

A possible explanation for the better performance of early-maturing dams is related to the efficiency of nutrient allocation. The lower feed conversion observed suggests that these birds may have a greater capacity to direct energy and nutrients toward egg formation, possibly due to differences in metabolic regulation or mitochondrial activity. Previous studies indicate that variations in mitochondrial efficiency can directly impact productivity in birds, influencing both energy metabolism and the production of reactive oxygen species (Hudson *et al*., 2017; Bottje, 2019).

When comparing the two groups of dams regarding immune parameters (Table 2), it was observed that the dams classified as early-maturing presented a lower percentage of heterophils (44.65% *vs.* 50.87%; p = 0.03918), a lower heterophil:lymphocyte ratio (0.824 *vs.* 1.050; p = 0.02747), and a lower relative spleen weight (0.027% *vs.* 0.046%; p = 0.03855) than the late-maturing dams. In contrast, late-maturing dams showed a higher percentage of lymphocytes than early-maturing dams (55.375% *vs*. 49.125%; p = 0.03918).

**Table 2:** Immunological parameters of Japanese quails classified as early-maturing or late-maturing.

Dams	Heterophils (%)	Lymphocytes (%)	H/L	Relative spleen weight (%)
Early	44.625^b^ ±6.28	55.375^a^ ±6.28	0.824^b^ ±0.18	0.027^b^ ±0.01
Late	50.875^a^ ±4.58	49.125^b^ ±4.58	1.050^a^ ±0.18	0.046^a^ ±0.02
p-value	0.03918	0.03918	0.02747	0.03855

^a,b^Different letters in the same column indicate statistical significance based on the t-test with a p-value <0.05.

Regarding the immunological parameters, the differences observed between the groups suggest a possible imbalance in the physiological state of the late-maturing dams. The higher proportion of heterophils and the increase in the heterophil:lymphocyte ratio are frequently associated with chronic stress conditions in birds (Scanes, 2016), which may indicate greater activation of the hypothalamic–pituitary–adrenal axis. In this context, the higher H/L value in the late-maturing dams may be related to a less favorable physiological state, potentially contributing to lower productive performance.

Furthermore, the greater relative spleen weight observed in late-maturing dams may indicate higher immune system activity or greater exposure to antigenic stimuli. Although an increase in lymphoid organ size may reflect greater immune capacity, it may also represent a significant energetic cost, as immune system activation demands resources that could be directed toward production (Conceição *et al*., 2026). However, it is important to consider that organ relative weight alone does not allow inferences about immune functionality, and caution is required when interpreting these results.

With respect to progeny performance, it was observed that offspring from early-maturing dams had a higher weight at 25 days of age (130 g *vs.* 124 g; p = 0.00961) and better feed conversion (5.7 *vs.* 6.5; p = 0.04235) than offspring from late-maturing dams. No significant differences were found for birth weight (p = 0.16381) (Table 3).

**Table 3:** Performance of daughters of early-maturing or late-maturing dams.

Dams	Birth weight	Weight at 25 days	Feed conversion
Early	8.099±0.65	130.273^a^±5.68	5.777^a^±0.65
Late	7.749±0.53	124.432^b^±3.68	6.537^b^±0.81
p-value	0.16381	0.00961	0.04235

^a,b^Different letters in the same column indicate statistical significance based on the t-test with a p-value <0.05.

The effects observed in the progeny reinforce the hypothesis that maternal precocity can influence post-hatch performance, possibly through maternal programming mechanisms (Abdel-Moneim, Shehata and Paswan, 2023). The better feed conversion and higher weight at 25 days in descendants of early-maturing dams are consistent with studies showing that egg quality, including nutrient composition, hormones, and immune factors, can affect the early development of birds (Kpodo and Proszkowiec-Weglarz, 2023). The absence of differences in birth weight suggests that these effects may not manifest during early embryogenesis but rather during post-natal growth. This dissociation between initial weight and subsequent performance has also been described in the literature and may be related to post-hatch metabolic adjustments, including digestive efficiency and nutrient utilization (Vaccaro *et al*., 2024).

Overall, the progeny performance data indicate the presence of two distinct phenotypes. This distinction was also explored in response to the LPS challenge, and the results are presented across the different levels of analysis shown below.

### Transcriptome analysis of Japanese quail progeny

The comparative analysis of the jejunal transcriptomes revealed that the maternal group programs distinct inflammatory responses in the progeny upon the LPS challenge (Fig. 1). In the offspring of early-maturing dams (Fig. 1A), a predominance of transcriptional suppression was observed, with 19 genes significantly repressed in the presence of LPS. Among them, regulators of mitochondrial energy metabolism, such as GPD1 and transporters of the SLC25 family (*SLC25A13* and *SLC25A15*), as well as DEPTOR and *IL4I1*, stand out. On the other hand, the genes expressed in the presence of LPS are mostly associated with the modulation of inflammation and cell communication, including *IL1R2, IL18BP, S100A12*, and *PDCD1LG2*. This pattern is consistent with a tolerance phenotype, characterized by metabolic restraint and control of the inflammatory response.

**Fig. 1:**
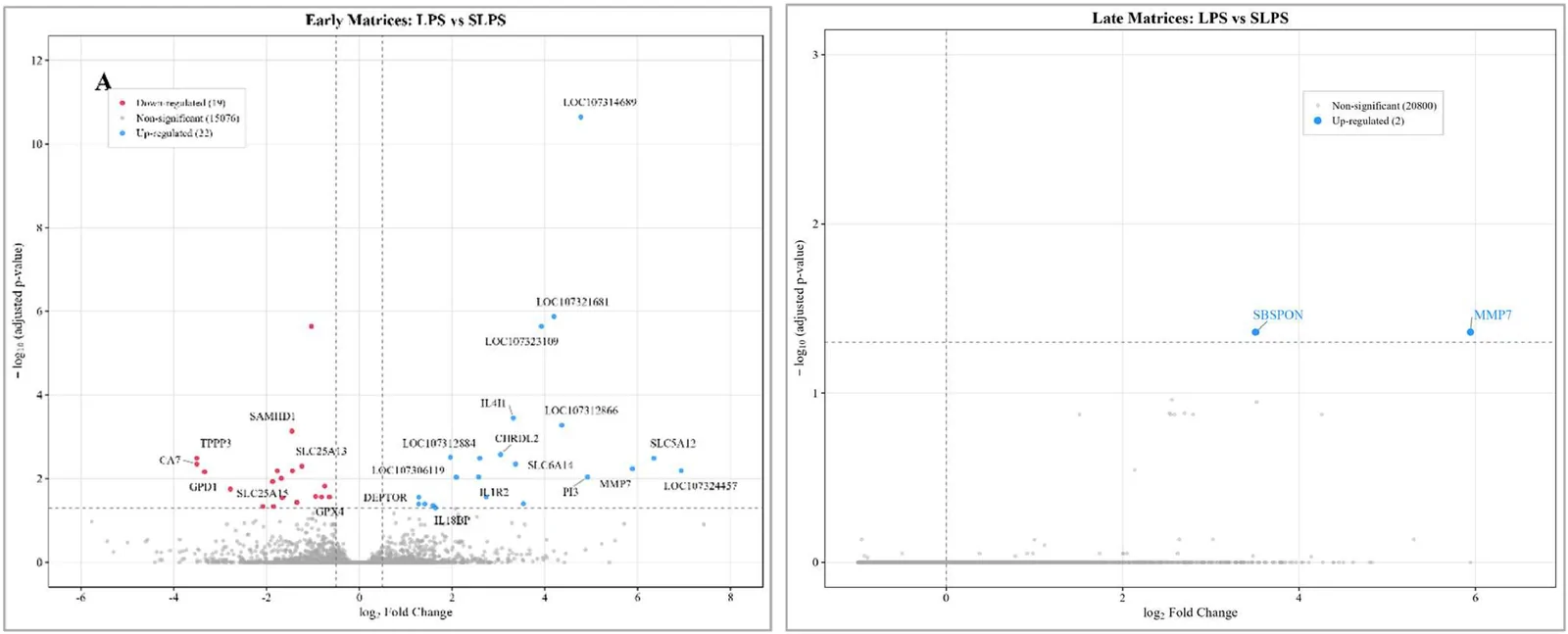
Volcano plots showing differential gene expression in the jejunum of quails from early-maturing (A) and late-maturing (B) dams challenged with LPS compared to respective controls (SLPS).

*Caption:* Volcano plots representing differential gene expression analysis in the jejunum of offspring from early-maturing (A) and late-maturing (B) dams after LPS challenge. Each point represents an individual gene. The x-axis indicates the magnitude of expression change (log2FoldChange) and the y-axis represents statistical significance (-log10 adjusted p-value). Horizontal dashed lines indicate the significance threshold (adjusted p = 0.05) and the vertical lines mark log2FoldChange = 0. Selected genes are identified by their symbols.

In contrast, the offspring of late-maturing dams (Fig. 1B) showed a profile of directed gene activation, with no genes significantly suppressed in the presence of LPS. The highlight was *MMP7*, with a high induction magnitude (log2FoldChange > 5), indicating activation of extracellular matrix remodeling processes in response to LPS.

### Functional enrichment analysis

The functional enrichment analysis also revealed that the maternal group significantly modulates the transcriptional profile of the progeny in response to the LPS-induced inflammatory challenge, evidencing two distinct biological strategies: a response of metabolic suppression with immune modulation in the offspring of early-maturing dams, and a response of tissue remodeling with proteolytic activity in the offspring of late-maturing dams.

## Offspring of early-maturing dams

The Biological Process analysis (GO:BP) of the genes suppressed in response to LPS (Table 4) demonstrated significant changes in metabolic and cellular transport pathways. Among the most significantly enriched terms, processes associated with the metabolism of nucleotides and redox cofactors stood out, including NADH metabolic process and NAD metabolic process, involving mainly the genes *GPD1* and *SLC25A13*. Enrichment of terms related to purine nucleotide metabolism and organophosphate catabolic processes was also observed, involving the genes *GPD1, SAMHD1*, and *SLC25A13*. Additionally, terms of transmembrane transport of l-amino acids and organic acids were identified, associated with the genes *SLC25A13* and *SLC25A15*.

**Table 4:** Genes suppressed in quails from early-maturing dams and inoculated with LPS.

ID GO: BP	Description	p.adjust	geneID
GO:0006734	NADH metabolic process	0.005157777	*GPD1/SLC25A13*
GO:1902475	L-alpha-amino acid transmembrane transport	0.005157777	*SLC25A13/SLC25A15*
GO:0015807	L-amino acid transport	0.006649841	*SLC25A13/SLC25A15*
GO:0019674	NAD metabolic process	0.006649841	*GPD1/SLC25A13*
GO:1905039	carboxylic acid transmembrane transport	0.012110144	*SLC25A13/SLC25A15*
GO:1903825	organic acid transmembrane transport	0.012110144	*SLC25A13/SLC25A15*
GO:0006163	purine nucleotide metabolic process	0.012110144	*GPD1/SAMHD1/SLC25A13*
GO:0003333	amino acid transmembrane transport	0.01570668	*SLC25A13/SLC25A15*
GO:0009117	nucleotide metabolic process	0.015959853	*GPD1/SAMHD1/SLC25A13*
GO:0019362	pyridine nucleotide metabolic process	0.015959853	*GPD1/SLC25A13*
GO:0046496	nicotinamide nucleotide metabolic process	0.015959853	*GPD1/SLC25A13*
GO:0072524	pyridine-containing compound metabolic process	0.015959853	*GPD1/SLC25A13*
GO:0006865	amino acid transport	0.015959853	*SLC25A13/SLC25A15*
GO:0072521	purine-containing compound metabolic process	0.015959853	*GPD1/SAMHD1/SLC25A13*
GO:0006753	nucleoside phosphate metabolic process	0.030370635	*GPD1/SAMHD1/SLC25A13*
GO:0055086	nucleobase-containing small molecule metabolic process	0.035891205	*GPD1/SAMHD1/SLC25A13*
GO:0046434	organophosphate catabolic process	0.035891205	*GPD1/SAMHD1*
GO:1901136	carbohydrate derivative catabolic process	0.035891205	*GPD1/SAMHD1*
GO:0046942	carboxylic acid transport	0.037142535	*SLC25A13/SLC25A15*
GO:0015849	organic acid transport	0.037142535	*SLC25A13/SLC25A15*

The GO:BP analysis of the genes suppressed in response to LPS indicated enrichment of pathways related to NAD/NADH metabolism, nucleotides, and amino acid transport, involving mainly *GPD1, SLC25A13*, and *SLC25A15*. This pattern suggests a metabolic reprogramming aimed at reducing mitochondrial activity and oxidative flux.

The decrease in the activity of *GPD1*, a component of the glycerol-3-phosphate shuttle, indicates a lower transfer of reducing equivalents to the mitochondria, which can affect oxidative phosphorylation and the production of reactive oxygen species (Sato *et al*., 2016). This adjustment may favor energy conservation and the minimization of oxidative damage during inflammation, a classic mechanism described from the perspective of immunometabolism (O’Neill *et al*., 2016).

In parallel, the Cellular Component analysis (GO:CC) of the genes expressed in this same group (Table 5) revealed an association with the cell surface and the extracellular environment. The term cell surface was significantly enriched, involving the genes *PDCD1LG2* and *ZPLD1*, indicating a possible intensification of cell-environment communication mechanisms. Complementarily, enrichment of terms related to extracellular vesicles, such as extracellular exosome and extracellular vesicle, associated with the *S100A12* gene, was observed.

**Table 5:** Genes expressed in quails from early-maturing dams and inoculated with LPS.

ID GO:CC	Description	p.adjust	geneID
GO:0009986	cell surface	0.04410568	*PDCD1LG2/ZPLD1*
GO:0070062	extracellular exosome	0.04410568	*S100A12*
GO:0043230	extracellular organelle	0.04410568	*S100A12*
GO:0065010	extracellular membrane-bounded organelle	0.04410568	*S100A12*
GO:1903561	extracellular vesicle	0.04410568	*S100A12*

The analysis of Molecular Functions (GO:MF) of the differentially regulated genes (Table 6) corroborated this functional duality. While the l-amino acid transport activity (*SLC25A13* and *SLC25A15*) was suppressed in the presence of LPS, significant expression of the terms cytokine binding (involving the genes *IL18BP* and *IL1R2*) and symporter activity (*SLC13A1* and *SLC5A12*) was observed.

**Table 6:** Differentially regulated genes in quails from early-maturing dams and inoculated with LPS.

ID GO:MF	Description	p.adjust	geneID
**UP**			
GO:0019955	cytokine binding	0.03226398	*IL18BP/IL1R2*
GO:0015293	symporter activity	0.0436887	*SLC13A1/SLC5A12*
**Down**			
GO:0015179	L-amino acid transmembrane transporter activity	0.03825773	*SLC25A13/SLC25A15*

The contrast between internal metabolic suppression and increased extracellular signaling suggests a state of silent alert in LPS-inoculated birds. The increase in the expression of genes associated with exosomes (*S100A12*) and the cell surface suggests an intensification of intercellular communication, a central mechanism in the regulation of the immune response (Yáñez-Mó *et al*., 2015). Crucially, the enrichment of the cytokine-binding function, particularly involving *IL18BP* (IL-18 binding protein) and *IL1R2* (IL-1 decoy receptor), indicates a negative modulation of inflammation. These molecules act by limiting the intensity of the inflammatory response through the sequestration of pro-inflammatory agonists (Dinarello, 2018). Together, these results indicate that the offspring of early-maturing dams adopt a stress tolerance strategy, characterized by an economical metabolism and fine control of the immune response, minimizing collateral tissue damage.

## Offspring of late-maturing dams

In opposition to the profile observed in the early-maturing dams, the functional enrichment analysis of the late-maturing dam offspring challenged with LPS revealed a strong and exclusive association with extracellular matrix remodeling processes, with all enriched terms related to the *MMP7* gene (Table 7).

**Table 7:** Genes expressed in quails from late-maturing dams and inoculated with LPS.

Category	ID GO	Description	p.adjust	geneID
**Biological Process**	GO:0030574	collagen catabolic process	0.003835663	*MMP7*
	GO:0032963	collagen metabolic process	0.003835663	*MMP7*
	GO:0030198	extracellular matrix organization	0.005710876	*MMP7*
	GO:0045229	external encapsulating structure organization	0.005710876	*MMP7*
	GO:0043062	extracellular structure organization	0.005710876	*MMP7*
**Cellular Component**	GO:0031012	extracellular matrix	0.01504859	*MMP7*
	GO:0030312	external encapsulating structure	0.01504859	*MMP7*
**Molecular Function**	GO:0004222	metalloendopeptidase activity	0.0249076	*MMP7*
	GO:0008237	metallopeptidase activity	0.0249076	*MMP7*
	GO:0004175	endopeptidase activity	0.03203171	*MMP7*
	GO:0008233	peptidase activity	0.03567411	*MMP7*

In the Biological Process category (GO:BP), significant enrichment was observed for terms such as collagen catabolic process, collagen metabolic process, and extracellular matrix organization. In Cellular Components (GO:CC), the terms extracellular matrix and external encapsulating structure were predominant. As for Molecular Functions (GO:MF), terms associated with proteolytic activity, such as metalloendopeptidase activity and peptidase activity, were identified.

These results reflect a more reactive and structural response to the inflammatory challenge. Matrix metalloproteinases (MMPs), specifically *MMP7*, are zinc-dependent endopeptidases widely recognized for their role in the degradation of collagen and other extracellular matrix components during tissue repair and inflammation processes (Ren *et al*., 2018). The expression of *MMP7* suggests that the offspring of late-maturing dams mount a response focused on tissue reorganization and cell migration in the face of LPS-induced damage. Although essential for repair, this type of response is energetically costly and, if exacerbated, may be associated with tissue damage and chronic inflammation (Bonnans *et al*., 2014). Unlike the offspring of early-maturing dams, which appear to “tolerate” stress by reducing metabolism, the offspring of late-maturing dams seem to actively “resist” through structural remodeling.

### KEGG pathway enrichment analysis

The functional enrichment analysis (Over-Representation Analysis – ORA) and Gene Set Enrichment Analysis (GSEA) revealed distinct metabolic and signaling profiles between the progenies, corroborating the different strategies of response to the inflammatory challenge programmed by the maternal group.

In the offspring of early-maturing dams (Table 8 – ORA), the LPS challenge resulted in the significant enrichment (adjusted p < 0.05) of pathways associated with lipid and amino acid metabolism, with emphasis on Arachidonic Acid Metabolism (*GPX4, PTGR1*) and Phenylalanine, Tyrosine and Tryptophan Biosynthesis (*IL4I1*), in addition to the general clustering of Metabolic Pathways. The GSEA (Table 9 – GSEA) deepened this view by demonstrating a coordinated suppression (DOWN-regulated) of central pathways of energy metabolism and redox homeostasis, including Oxidative Phosphorylation (*NDUFA5, NDUFS6*), Pyruvate Metabolism (*ACOT12, ACACA*), Fatty Acid Metabolism (*ALDH3A2, ACAT1*), and Glutathione Metabolism (*GPX1, GSTZ1*). Additionally, suppression of the Cell Cycle (*CDC25A, CCNE1*) was observed. In contrast, pathways of protein synthesis (Ribosome) and steroid biosynthesis were activated.

**Table 8:** Functional Enrichment Analysis of offspring from early- or late-maturing dams inoculated with LPS.

ID KEGG	Description	p.adjust	geneID
**Early-maturing dams**			
KEGG:00000	KEGG root term	0.008473	*ACOT12/CA7/DEPTOR/GPX4/IL1R2/IL22/IL4I1/PDCD1LG2/ACOD1L/MFN1/MMP7/NDUFA5/PTGR1/RHOB/SAMHD1*
KEGG:00590	Arachidonic acid metabolism	0.015744	*GPX4/PTGR1*
KEGG:00400	Phenylalanine, tyrosine and tryptophan biosynthesis	0.029013	*IL4I1*
KEGG:01100	Metabolic pathways	0.029013	*ACOT12/CA7/GPX4/IL4I1/ACOD1L/NDUFA5*
**Late-maturing dams**			
KEGG:04310	Wnt signaling pathway	0.003387	*MMP7*

**Table 9:** GSEA comparing early-descendants challenged with LPS vs. early-descendants sham (SLPS).

KEGG Pathway	NES*	p-adjust	Gene IDs
**Activated Pathways (UP-regulated)**			
Ribosome	2.185	0.006	*RPL12/ RPL8/ RPS26*
Steroid Biosynthesis	2.160	0.006	*DHCR24/ FDFT1/ SQLE*
Leukocyte Transendothelial Migration	2.011	0.006	*NOX1/ CXCR4/ PLCG1*
Cytokine-cytokine Receptor Interaction	1.829	0.013	*IL1R2/ IL22/ IL18*
Jak-STAT Signaling Pathway	1.627	0.037	*IL22/ IL21R/ IL13RA2*
**Suppressed Pathways (DOWN-regulated)**			
Oxidative Phosphorylation	−2.098	0.006	*NDUFA5/ ATP12A/ NDUFS6*
Cell Cycle	−1.978	0.006	*CDC25A/ ORC3/ CCNE1*
Propanoate Metabolism	−1.913	0.010	*ACACA/ ALDH3A2/ ACAT1*
Pyruvate Metabolism	−1.866	0.032	*ACOT12/ DLAT/ ACACA*
Glutathione Metabolism	−1.837	0.034	*MGST2/ GSTZ1/ GPX1*
Fatty Acid Metabolism	−1.814	0.037	*ALDH3A2/ ACAT1/ ACAA1*
Lysosome	1.566	0.043	*LAPTM4B/ ATP6V0D2/ LAPTM5*

⁎ NES: Normalized Enrichment Score. Positive values indicate pathway activation in the LPS-treated group; negative values indicate suppression.

In contrast, the offspring of late-maturing dams challenged with LPS presented a markedly distinct and more restricted functional enrichment profile. The ORA exclusively identified the Wnt Signaling Pathway (*MMP7*) as significantly enriched (adjusted p = 0.003).

The pathway enrichment analysis corroborated the observed patterns. In the offspring of early-maturing dams, ORA indicated the enrichment of metabolic pathways, including arachidonic acid metabolism and biosynthesis of aromatic amino acids. GSEA revealed coordinated suppression of central energy metabolism pathways, such as oxidative phosphorylation, pyruvate metabolism, fatty acid metabolism, and glutathione metabolism, in addition to the cell cycle.

This pattern indicates a functional shutdown of the main ATP-producing pathways, consistent with a metabolic tolerance strategy. The reduction in mitochondrial respiratory chain activity suggests lower generation of reactive oxygen species, a mechanism widely described as a means to limit inflammatory damage (O’Neill *et al*., 2016). The suppression of the cell cycle indicates prioritization of cellular maintenance over proliferation. In contrast, the activation of pathways such as ribosome suggests energetic redirection toward specific protein synthesis, possibly associated with the immune response.

In the offspring of late-maturing dams, exclusive enrichment of the Wnt signaling pathway was observed. This pathway acts as a central regulator of cell proliferation and intestinal epithelial renewal (Clevers and Nusse, 2012). Together with the induction of *MMP7*, these results indicate the activation of a functional axis aimed at tissue remodeling and structural response to damage.

### Maternal programming: phenotypic and molecular integration

The integrated analysis of the data indicates that maternal precocity is associated with the programming of distinct physiological strategies in the progeny in response to the inflammatory challenge. Instead of isolated differences, the results point to two organized modes of response, involving coordination between metabolism, immune system, and productive performance.

In the offspring of early-maturing dams, the body of evidence is consistent with a strategy of regulating the inflammatory response coupled with energy expenditure restraint. The modulation of immune mediators with regulatory function, combined with the redirection of metabolic pathways, suggests a fine adjustment between immune activation and the preservation of cellular homeostasis. This type of response is compatible with mechanisms described in the context of immunometabolism, in which changes in energy metabolism act as modulators of the intensity of inflammation (O’Neill *et al*., 2016). Functionally, this arrangement may contribute to greater nutrient utilization efficiency, reducing costs associated with the immune response and favoring the maintenance of performance.

On the other hand, the offspring of late-maturing dams exhibited a pattern compatible with a more reactive and structural response to the inflammatory stimulus. The activation of processes related to extracellular matrix dynamics and tissue reorganization indicates a direction of the response toward damage containment and repair. Although effective in acute contexts, this type of response is associated with greater energy demand and may imply trade-offs with productive functions, especially when sustained over time.

These differences suggest that the maternal condition not only influences progeny performance, evaluated in this study during the growth phase, but also defines the way organisms allocate resources in the face of physiological challenges. In addition to the possible explanation already reported in the literature based on egg composition, including hormones and other bioactive factors capable of modulating metabolic and immunological development (Reynolds *et al*., 2019; Park and Sohn, 2018), our work provides new information based on genetic differences which, reflected in the phenotypic differences between dams, act as a direct determinant of the progeny’s responses.

From an adaptive perspective, the two identified profiles may represent complementary strategies for different environmental scenarios. While one favors tolerance and resource economy, the other prioritizes faster and more structural responses to damage. Understanding these patterns contributes to elucidating the effects of maternal origin on productive efficiency and physiological resilience in birds.

## Conclusion

The functional analysis confirms that the female’s productive efficiency, characterized by the groups of early-maturing and late-maturing dams, is a determining factor in the programming of the progeny’s immune response, dictating a binary choice between tolerance (energy conservation) and resistance (tissue remodeling) to the challenge caused by LPS inoculation.

## Fouding sources

This study was financed by the Brazilian National Council for Scientific and Technological Development (CNPq. PROC. 403205/2021-2). The Brazilian Federal Agency for Support and Evaluation of Graduate Education (CAPES. Financial code 001).

## Author contributions

Juliana dos Santos Conceição conceived the study together with the supervisors, conducted the animal trial, performed the bioinformatic and statistical analyses, interpreted the productive, immunological, and transcriptomic data, integrated the findings, and drafted the manuscript. Eliane Gasparino and Ana Paula del Vesco conceived and supervised the study, designed the experimental approach, guided the interpretation of the biological and transcriptomic findings, and critically revised the manuscript. Simara Márcia Marcato contributed to the experimental design, supervised the nutritional aspects of the study, participated in the interpretation of the results, and critically revised the manuscript. Angélica de Souza Khatlab contributed to the molecular analyses, participated in the bioinformatic interpretation of the transcriptomic data, and critically revised the manuscript. Marcos Adriano Pereira Barbosa, Vanicleide da Silva Santos, Lucas Soares da Silva, Marisa Silva Bastos, Thais Pacheco Santana, and Emine Mayer Chamse Ddine participated in conducting the experiment and laboratory analyses, contributed to data interpretation, and critically discussed the results. Vittor Tuzzi Zancanella, Claudson Oliveira Brito, and Jodnes Sobreira Vieira contributed to the scientific interpretation of the findings, participated in the discussion of the results, and critically revised the manuscript.

## Disclosures

The authors declare that there are no conflicts of interest regarding the publication of this manuscript.
